# A Molecular Assay for Sensitive Detection of Pathogen-Specific T-Cells

**DOI:** 10.1371/journal.pone.0020606

**Published:** 2011-08-11

**Authors:** Victoria O. Kasprowicz, Jessica E. Mitchell, Shivan Chetty, Pamla Govender, Kuan-Hsiang Gary Huang, Helen A. Fletcher, Daniel P. Webster, Sebastian Brown, Anne Kasmar, Kerry Millington, Cheryl L. Day, Nompumelelo Mkhwanazi, Cheryl McClurg, Fundisiwe Chonco, Ajit Lalvani, Bruce D. Walker, Thumbi Ndung'u, Paul Klenerman

**Affiliations:** 1 Ragon Institute of MGH, MIT and Harvard, Harvard Medical School, Boston, Massachusetts, United States of America; 2 Kwazulu-Natal Research Institute for TB and HIV, Nelson R. Mandela School of Medicine, Durban, South Africa; 3 HIV Pathogenesis Programme, Doris Duke Medical Research Institute, Nelson R. Mandela School of Medicine, Durban, South Africa; 4 Nuffield Department of Medicine, The Jenner Institute, University of Oxford, Oxford, United Kingdom; 5 Nuffield Department of Medicine, Oxford Biomedical Research Centre and James Martin School for 21st Century, University of Oxford, Oxford, United Kingdom; 6 Division of Rheumatology, Immunology and Allergy, Brigham and Women's Hospital, Harvard Medical School, Boston, Massachusetts, United States of America; 7 Tuberculosis Research Unit, Department of Respiratory Medicine, National Heart and Lung Institute, Imperial College London, London, United Kingdom; Institut de Pharmacologie et de Biologie Structurale, France

## Abstract

Here we describe the development and validation of a highly sensitive assay of antigen-specific IFN-γ production using real time quantitative PCR (qPCR) for two reporters - monokine-induced by IFN-γ (MIG) and the IFN-γ inducible protein-10 (IP10). We developed and validated the assay and applied it to the detection of CMV, HIV and Mycobacterium tuberculosis (MTB) specific responses, in a cohort of HIV co-infected patients. We compared the sensitivity of this assay to that of the ex vivo RD1 (ESAT-6 and CFP-10)-specific IFN-γ Elispot assay. We observed a clear quantitative correlation between the two assays (P<0.001). Our assay proved to be a sensitive assay for the detection of MTB-specific T cells, could be performed on whole blood samples of fingerprick (50 uL) volumes, and was not affected by HIV-mediated immunosuppression. This assay platform is potentially of utility in diagnosis of infection in this and other clinical settings.

## Introduction

T cells provide a critical immune response against many infections, including persistent pathogens such as human immunodeficiency virus (HIV), cytomegalovirus (CMV) and Mycobacterium tuberculosis (MTB). The measurement of T cell responses has undergone a revolution in the last decade with the emergence of novel *ex vivo* reagents such as MHC peptide tetramers and functional assays such as interferon-gamma (IFN-γ)-Elispot [1]. These assays have allowed accurate quantification of such responses in acute and persistent infections and after vaccination. Contemporaneously, real time quantitative PCR (qPCR) has become an important method for profiling the transcriptional states of cells and tissues. Moreover, qPCR has proved to be a novel, promising method for the monitoring of cytokine release in effector T cells against tumour and pathogen antigens [2].

IFN-γ secreted by stimulated CD8+ and CD4+ T cells acts upon other cell types, including monocytes and neutrophils, which respond through gene upregulation [3]. Since IFN-γ responsive monocytes and neutrophils are more numerous in peripheral blood than antigen specific T cells (approximately 1000–10,000 fold), IFN-γ-induced gene expression provides an important amplification step for the IFN-γ signal. We reasoned that this “self-amplifying” RNA signal might provide a more sensitive detection method for antigen exposure than measuring IFN-γ production directly. Two such reporters of IFN-γ production are monokine-induced by IFN-γ (MIG) and the IFN-γ inducible protein-10 (IP10). They are members of the CXC chemokine family and are predominantly produced by cells of the monocyte/macrophage lineage bearing CD14. They are involved in trafficking monocytes and activated Th1 cells to inflamed foci through interaction with their common CXCR3 chemokine receptor. MIG has also been shown to stimulate T lymphocyte proliferation and effector cytokine production in addition to its chemotactic effects. Secretion of MIG is induced by interferon via the janus kinase–signal transducer and activator of transcription (JAK-STAT) pathway. In contrast to other interferon-stimulated genes, optimal induction of IP10 is dependent on activation of p38 kinases and both MIG and IP10 are induced by IFN-γ as well as TNF-alpha. Importantly, both MIG and IP10 are expressed in high quantities in an antigen-specific manner following stimulation with antigens from a variety of pathogens, including CMV and MTB [4], [5]. Such analyses have used Northern blotting, ELISA, Elispot, flow cytometry, and multiplex to measure IP10 and MIG concentration in patient serum or after in vitro stimulation with antigens. Subsequently, IP10 and MIG were proposed as adjunct biomarkers for tuberculosis (TB). However, measuring MIG and IP10 production with these techniques did not reach the sensitivity of the standard IFN-γ Elispot [5], [6], [7], [8]. Interestingly, a recent study in active tuberculosis has shown that measuring IP10 by Elisa in addition to IFN-γ increases diagnostic sensitivity [9], [10]. Real time qPCR has been used frequently to measure the antigen-specific immune responses of very small populations of cells and is gaining popularity in vaccine immunology [11]. A recent study used qPCR for MIG to detect responses in separated PBMCs to vaccines in a controlled setting [3] and has shown that MIG expression may correlate with protection against malaria [12]. It is a versatile tool and can be used to measure the expression of virtually any mRNA transcript. We therefore sought to develop a novel qPCR-based assay for MIG and IP10 for the detection of pathogen-specific T cells and apply this to the detection of MTB infection, as an example of its application.

## Materials and Methods

Ethical approval and informed consent was obtained from all individuals who participated in this study (UKZN ethics board, McCord Hospital ethics board, Massachusetts General Hospital Ethics Board, University of Oxford ethics board and Imperial College London ethics board).

### qPCR Assay Development

PBMCs from healthy, CMV infected volunteers were used to establish the qPCR assay for the quantification of MIG and IP10 expression. Recombinant human IFN-γ was used as a positive control to induce MIG and IP10 expression in ex vivo PBMCs, as IFN-γ production in vivo is a potent inducer of both MIG and IP10 expression. Whole cell CMV lysate was used as an antigenic stimulant. 10^6^ fresh PBMCs/condition were stimulated with 10 ng/mL IFN-γ or 10 µL/ml CMV whole cell lysate (Sigma) for 8, 12 and 16 hours. 10 µL R10 media was used as a negative control. 10^6^ PBMCs/condition were stimulated with 10 ng/mL IFN-γ (positive control), 10 µL/mL of CMV whole cell lysate (Sigma), dilutions of CMV whole cell lysate (1∶10, 1∶100, or 1∶1,000) for 16 hours or 10 µL R10 (null). After stimulation, mRNA was extracted and processed by reverse transcription and qPCR as described below.

### mRNA Extraction

Extraction of cellular mRNA as performed using an RNeasy mini kit (Qiagen) as per the manufacturer's instructions. The stimulation of PBMCs was stopped at 16 hours by lysing the cells with 350 µL RLT buffer with 1% β-mercaptoethanol. QIAShredder® (Qiagen) spin columns were used to homogenize the PBMCs before mRNA extraction. All extracted mRNA was eluted in 35 µl of RNase-free water and stored at −80°C.

### Reverse transcriptase and RT-Quantitative PCR

Extracted cellular mRNA was reverse transcribed to cDNA using iScript cDNA synthesis kit (BIO-Rad) and 10 uL of cellular mRNA. All RT-PCR was performed on an Applied BioSystems GeneAmp® PCR System970 per the manufacturer's instructions. cDNA was stored at −20°C until qPCR was performed.

After stimulation, mRNA extraction and cDNA transcription, real-time qPCR was performed for MIG, IP10, and HPRT using SYBR Green Master Mix (Qiagen) for a total volume of 20 µL; 1 µL input cDNA was used. Primer sequences, concentrations of forward and reverse primers, and the size of the gene amplicons are given:

**MIG:** 5′-GTG GTG TTC TTT TCC TCT TG-3′, 5′-GTA GGT GGA TAG TCC

CTT GG-3′, 0.5 pmol/µL, 120 bp;

**IP10:** 5′-TGA TTT GCT GCC TTA TCT TTC TGA-3′; 5′-CAG CCT CTG TGT

GGT CCA TCC TTG-3′, 0.25 pmol/µl, 408 bp;

**HPRT:** 5′-TAG GAC AGG ACT GAA CGT C-3′, 5′-CTA CAA TGT GAT GGC

CTC CC-3′, 0.5 pmol/µl, 64 bp.

RT-qPCR parameters for IP10 were 10 minutes at 95°C, and 45 cycles of 95°C, 61°C and 72°C at 10 seconds each. MIG and HPRT parameters were 10 minutes at 95°C and 45 cycles of 16 seconds at 95°C, 10 seconds at 59°C and 10 seconds at 72°C.

Standard curves for each gene of interest, cDNA for HPRT, MIG, and IP10 were created via RT-PCR as described. The amplified cDNA samples were purified by QIAquick PCR Purification (Qiagen) according to the manufacturer's directions and then quantified by UV spectrophotometry with NanoDrop 1000 (ThermoScientific). The number of cDNA copies was calculated using the molecular weight of each gene amplicon and the formula: RNA copy number  =  moles DNA ×6.02×10^23^. The samples were then serially diluted and tested by qPCR under the defined conditions. All realtime qPCR reactions were performed in a 96-well optical microtiter plate (Roche) using SYBR Green PCR Master Mix and a LightCycler®480 II system (Roche). All reactions were completed in duplicate and reported as the average. MIG and IP10 expression were normalized to HPRT expression, and the fold increase of each was calculated using the formula: Fold increase  =  ((MIG or IP10 stimulated / MIG or IP10 unstimulated) / (HPRT stimulated/ HPRT unstimulated)).

### CMV IFN-γ Elispot, MTB Region of Difference-1 (RD1) IFN-γ Elispot and HIV IFN-γ Elispot

96-well polyvinylidene difluoride-backed Elispot plates (MAIP S45, Millipore) were coated overnight at 4°C with 100 µl anti-IFN-γ antibody (1-D1k, 0.5 µg/ml, MabTech, Sweden). The plates were then washed six times with blocking buffer (1% fetal calf serum (FCS) in PBS). 50 µl of R10 (RPMI 1640 medium supplemented with 10% FCS, 1% L-glutamine, and 1% penicillin/streptomycin) were added to the empty wells. CMV, MTB Region of Difference-1 (RD1) or HIV antigens were then added to each well. For the CMV Elispot, 200,000 cells/well were stimulated with CMV lysate (10 ug/ml) (Sigma), serially diluted 1, 1∶10, 1∶100, or 1∶1,000, and incubated overnight at 37°C and 5% CO_2_. All CMV stimulations were performed in duplicate and reported as the average. For the RD1 Elispot, ESAT-6 and CFP-10 peptide pools were added at a final peptide concentration of 8 ug/ml. For the HIV Elispot, HIV peptide pools were added at a final concentration of 10 ug/ml. Phytohemagglutinin (PHA) (10 µg/ml) was used as a positive control. PBMC were plated at 200,000 cells/well and incubated overnight at 37°C and 5% CO_2_. All stimulations were performed in triplicate and reported as the average. The plates were then washed with PBS and 0.5 µg/ml of biotinylated anti-IFN-γ antibody (7-B6-1, MabTech) was added. The plates were washed with PBS then incubated with 0.5 µL/mL Streptavidin-alkaline phosphatase conjugate (Mabtech) for 45 minutes. After a final wash, IFN-γ producing cells were identified by direct visualization of spots produced by the addition of alkaline phosphatase colour reagents (Bio-Rad). CMV Elispot responses with a mean minus negative control >50 spot-forming cells (SFC)/ 10^6^ PBMCs were considered positive. RD1 and HIV- specific responses were considered positive if the number of spots per well was four SFC more than negative control wells and this number was at least twice that in negative control wells. This pre-defined cut-off point translates into a detection threshold of 20 peptide-specific T cells per million PBMC [13].

### Assay Verification

For the verification of the qPCR assay in a clinical setting, informed consent was obtained from 58 patients (HIV and TB therapy naïve, chronically infected with HIV-1 clade C) with a CD4 T cell count range of 96–996 cells/µl, in Durban, South Africa. Active TB was excluded by a symptom-screening questionnaire and physical examination. We used our own generic RD1 (ESAT-6 and CFP-10) based Elispot –performed on freshly isolated PBMC-to categorize patients as latently infected (Elispot positive) or uninfected (Elispot negative). 27 individuals were ex vivo RD1 Elispot positive (RD1 positive), indicating suspected latent MTB infection (there is no gold standard for the diagnosis of latent MTB infection). 31 individuals were ex vivo RD1-Elispot negative (RD1 negative), indicating absent MTB infection. qPCR was performed on frozen PBMC from these 46 study participants. qPCR was performed on fresh PBMC from 24 of these study participants. A matched comparison of MIG/IP10 expression between freeze-thawed versus fresh PBMCs was performed on a subset of 12 patients. Informed consent was also obtained from 32 HIV positive individuals with culture-confirmed TB with a CD4 count range of 17–734 cells/µl. qPCR was performed on frozen PBMC for 23 study participants with active TB and fresh PBMC from 9 study participants with active TB. PBMCs (10^6^ cells/condition) were stimulated with 10 ng/ml recombinant IFN-γ (positive control), 25 µl of R10 media (null), or ESAT-6 and CFP-10 peptide pools (final peptide concentration of 8 µg/ml) at 37°C and 5% CO_2_ for 16 hours. HIV gag and pol peptide pools were added at a final concentration of 10 ug/ml. If frozen, PBMCs were thawed and rested for 2 hours before stimulation. mRNA extraction, RT and qPCR was performed as described above. RD1 positive and RD1 negative individuals, and active TB patients were matched for CD4 count; differences in CD4 count were not statistically significant.

In order to establish a cut-off the qPCR assay was performed on PBMC from 9 HIV negative individuals who were all RD1 Elispot negative and negative by PPD skin test (negative controls). A test assay cut-off of mean + 3 Standard Deviations (SD) was used (MIG ESAT-6 = 1.5361; MIG CFP-10 = 2.9748; IP10 ESAT-6 = 2.9244; IP10 CFP-10 3.1709). To be considered positive for MTB infection, MIG and IP10 expression in response to IFN-γ stimulation had to be greater than all of the cutoffs, AND be positive for ESAT-6 and/or CFP-10 by MIG and/or IP10. If MIG and IP10 production in response to IFN-γ were less than any of the cutoffs, the assay was considered to have failed for that chemokine/antigen combination.

### Whole Blood Miniaturization

100, 25 and 10 µL of whole blood from 3 healthy, CMV infected donors with was diluted 1∶5 with R10 media and stimulated with 10 ng/mL IFN-γ or ten-fold dilutions of CMV lysate (Sigma) for 16 hours. Whole blood from 8 HIV+, RD1 Elispot positive patients (25 or 50 µl of whole blood diluted 1∶5 in R10 media) was stimulated with 10 ng/ml recombinant IFN-γ (positive control), 25 ul of R10 media (null, negative control), or titrated ESAT-6 and CFP-10 peptides at 37°C and 5% CO_2_ for 16 hours. For the ESAT-6 and CFP-10 peptide titration, whole blood (25 µL or 50 µL diluted 1∶5 with R10 media) was stimulated with 1, 5, 10, or 20 µl of 0.16 mg/ml ESAT-6 and CFP-10 peptide pools resulting in a final individual peptide concentration of 1.33, 6.64, 13.28 and 26.56 µg/ml for 25 µl of whole blood and 0.66, 3.32, 6.64 and 13.28 µg/ml for 50 µl of whole blood. HIV gag and pol peptide pools were added at a final concentration of 10 ug/ml. Whole blood was processed using QIAamp ® RNA Blood mini kit (Qiagen) according to the manufacturer's instructions. All extracted mRNA was eluted in 35 ul of RNase-free water and stored at −80°C. RT and qPCR were performed as described above.

### Statistical Analysis

Differences between medians were determined by using the Mann-Whitney test for unpaired data and the Wilcoxon test for paired data. A Kruskal-Wallis test was used to compare three or more parameters. Selected pairs of data were analyzed with a Dunn's multiple comparison test. The correlation between two parameters was determined by calculating the Spearman correlation coefficient, *r*. All t-tests tests were two-tailed and a nonparametric distribution was assumed. P values where P*<*0.05 were considered significant.

## Results

### Development of a novel assay for the detection of antigen-specific T cells

**Figure 1:**
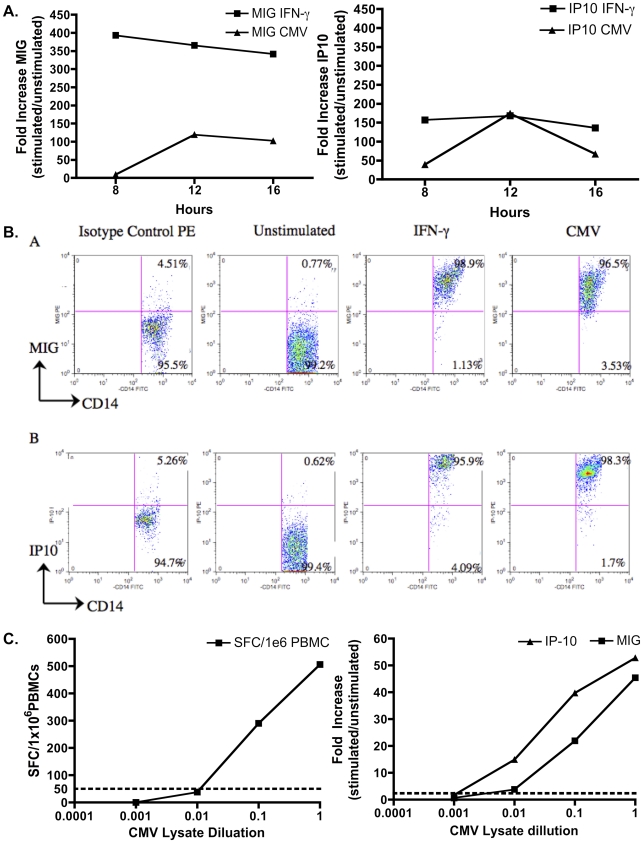
Development of a novel assay for the detection of antigen-specific T cells. a. Time course of cytokine-induced as compared to antigen-induced MIG and IP10 expression. qPCR analysis of MIG (left) or IP10 (right) mRNA obtained from PBMCs stimulated with 10 ng/mL IFN-γ or 10 µl/ml CMV whole cell lysate for 8, 12 and 16 hours. b. FACs analysis of MIG (top) and IP10 (bottom) in CD14+ Monocytes cultured for 16 hours with 10 ng/ml IFN-γ or 10 µl/ml CMV lysate. Monocytes were gated based on forward and side scatter. Cells were stained with mAb to CD14, fix and permeabilized, and stained with mAbs specific for MIG or IP10. Cells gated within the monocytes/macrophage regions were evaluated for MIG and IP10 expression. Isotype antibody for MIG and IP10 was used to control for non-specific binding. c. IFN-γ Elispot (left) and IP10/MIG qPCR (right) to serially diluted CMV lysate. The data shown represents that average of two independent experiments. Variation between experiments was insignificant, as measured by a Paired *t-*Test (P*>*0.05). The parallel line indicates a cut-off of 50SFC/10^6^ PBMCs for the IFN-γ Elispot (left). For qPCR (right), a test assay cut-off of mean + 3SD was used (MIG ESAT-6 = 1.5361; MIG CFP-10 = 2.9748; IP10 ESAT-6 = 2.9244; IP10 CFP-10 3.1709).

We initially developed and validated our qPCR assay system by the analysis of immune responses to CMV. CMV infection is a major health threat in those with impaired cellular immunity (including neonates, those receiving immunosuppression such as solid organ transplant (SOT) and haematopoetic stem cell transplant (HSCT) recipients and in HIV/AIDS) and provided a good model for chronic infection as most people have detectable responses [14]. To determine the timing of maximal MIG/IP10 mRNA production following stimulation, we cultured PBMCs from healthy donors with IFN-γ or CMV for 8, 12 or 16 hours. These experiments indicated that a 12–16 hour timepoint was optimal for detection of both MIG and IP10 (Figure 1a). To analyze the cellular source of the signals, we examined monocyte expression of MIG and IP10 via FACS under identical conditions. CD14+ monocytes were shown to strongly express MIG and IP10 when stimulated with CMV antigen (96.5% and 98.3% of CD14+ MIG+ and IP10+, respectively; figure 1b), confirming reports that macrophages and monocytes are the major MIG secreting cell populations [8], [15]

**Figure 2:**
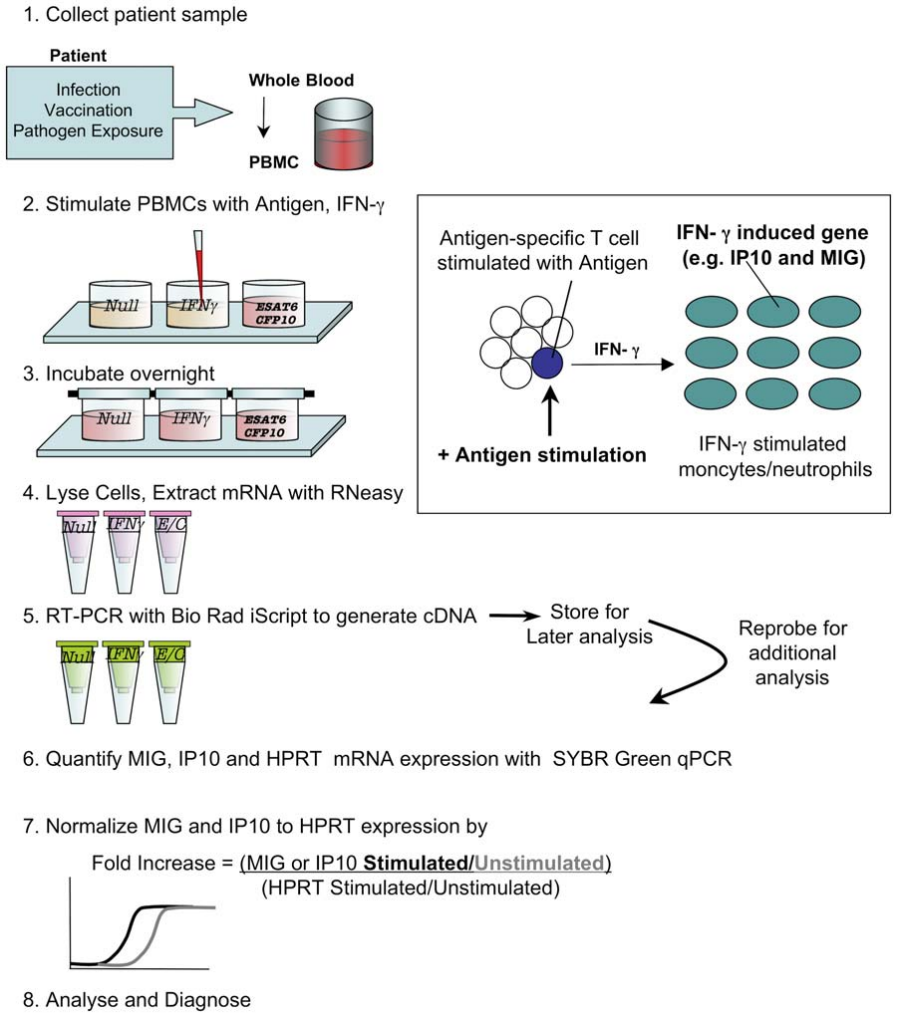
A molecular assay for sensitive detection of pathogen-specific T-cells. Fresh or frozen PBMCs or whole blood are stimulated overnight (16 hours) with 10 ng/ml IFN-γ, 8 µg/ml ESAT-6 and 8 µg/ml CFP-10 or nothing (null). After stimulation, the cells are lysed and mRNA extracted, cDNA synthesized via reverse transcriptase PCR. MIG, IP10 and HPRT mRNA expression are then quantified by quantitative real-time PCR. MIG and IP10 expression is normalized by HPRT, a housekeeping gene, as well as an internal negative control (Null). Fold increase is calculated by the formula: Fold increase  =  ((CXC stimulated / CXC unstimulated) / (HPRT stimulated/ HPRT unstimulated)). Importantly, the assay is highly flexible, can be stored at any stage for batching and is amenable to automation. A test assay cut-off of mean + 3SD was used (MIG ESAT-6 = 1.5361; MIG CFP-10 = 2.9748; IP10 ESAT-6 = 2.9244; IP10 CFP-10 3.1709). To be considered positive for MTB infection, MIG and IP10 expression in response to IFN-γ stimulation had to be greater than all of the cutoffs, AND be positive for ESAT-6 and/or CFP-10 by MIG and/or IP10.

We next compared the limit of detection of the qPCR assay in PBMCs to IFN-γ Elispot, by limiting responses through dilution of CMV antigen. The PBMC qPCR assay was able to detect MIG and IP10 expression at responder cell frequencies at least as low as those detected by the IFN-γ Elispot. Using cut-off values as defined in methods, the Elispot was negative for CMV antigen dilutions below 1 in 100, whereas the qPCR assay was still positive at 1 in 100 dilution (Figure 1c). In these experiments IP10 expression was significantly higher than MIG expression (*p* = 0.002) and provided a more sensitive measure of antigen reactivity. Analysis of T cell responses against CMV therefore provided a useful model in which to validate the assay as well as potentially providing a critical tool to monitor antiviral CMV responses in those at risk of disease. A schematic diagram of our qPCR assay is displayed in figure 2.

### MTB infection as an example application

We next wished to assess how the sensitivity of this assay platform for detection of responses to MTB region of difference-1 (RD1) antigens ESAT-6 and CFP-10 in a cohort of individuals with confirmed MTB and HIV co-infection. RD1 is a section of the genome that is present in MTB but deleted from all strains of BCG and most environmental mycobacteria. Initially we analysed three different cohorts of MTB/HIV coinfected individuals: patients with culture confirmed active TB (n =  23), latent MTB infection (n = 20), and MTB uninfected (n = 26; see methods). IFN-γ release assays (IGRAs) (T-SPOT®.TB, based on Elispot, and QuantiFERON®, based on ELISA technology) show increased sensitivity and specificity when compared to the traditional tuberculin skin test (TST) [16]. Unfortunately, TST sensitivity is greatly reduced in HIV co-infected individuals due to HIV-induced cutaneous anergy resulting in negative skin tests despite active mycobacterial infection [17], [18]. IGRAs are now used routinely in clinical care in the Western World, for example, in the USA, the CDC has recommended that the QuantiFERON-TB Gold replace the TST for the diagnosis of latent MTB infection [19]. We used our own in-house RD1 (ESAT-6 and CFP-10) based Elispot to categorize patients as latently infected or uninfected (in the absence of symptoms of active TB disease). Importantly, all the individuals used in this study were categorized based on having duplicate assay results at 2 time-points 3 months apart. All Elispots were performed on freshly isolated PBMC samples, and, subsequently, qPCR on frozen samples. Based on our assay platform an individual was considered qPCR positive if they had a ratio of stimulated:unstimulated expression for IP10 or MIG following antigenic stimulation exceeding 3SD from mean control values. For our choice of MTB antigens this means that an individual is considered positive by displaying reactivity to one or more of the following: ESAT-6 IP10, ESAT-6 MIG, CFP-10 IP10 and/or CFP-10 MIG. One-way ANOVA with Kruskal-Wallis test was p = 0.0012 for IP10/ESAT-6, p = 0.0005 for IP10/CFP-10, p = 0.0018 for MIG/ESAT-6, and p = 0.0013 for MIG/CFP-10. Dunn's Multiple comparison test was then performed. Our novel assay approach was able to differentiate between people that are RD1-Elispot negative (i.e. those we presume to be uninfected) and those we have categorized as latently infected (RD1 positive) (IP10/ESAT-6 p<0.001, IP10/CFP-10, p<0.01MIG/ESAT-6 p<0.01, and MIG/CFP-10 p<0.001) (data not shown). However, like the IGRAs, it is unable to differentiate between RD1 positive (latently MTB infected) and those with active TB disease.

The ability to use either fresh or frozen cells provides many technical advantages to our qPCR assay. However, many recent studies have reported that freeze-thawed PBMCs show altered gene expression, viability and functionality. To determine if freezing and thawing cells alters the expression of MIG and IP10, and if our assay and its sensitivity could potentially be improved by using fresh PBMCs, we performed our standard MTB assay on matched fresh and frozen samples. We then compared the levels of MIG and IP10 mRNA expression between the fresh and frozen sample groups (Wilcoxon matched pairs test). The mean MIG and IP10 expression was significantly higher in fresh PBMC samples as compared to frozen PBMCs (data not shown), indicating that testing fresh rather than frozen samples could significantly improve the MIG and IP10 signal (p =  0.00676, 0.0023, 0.0295 and 0.0040 for the four conditions).

**Figure 3:**
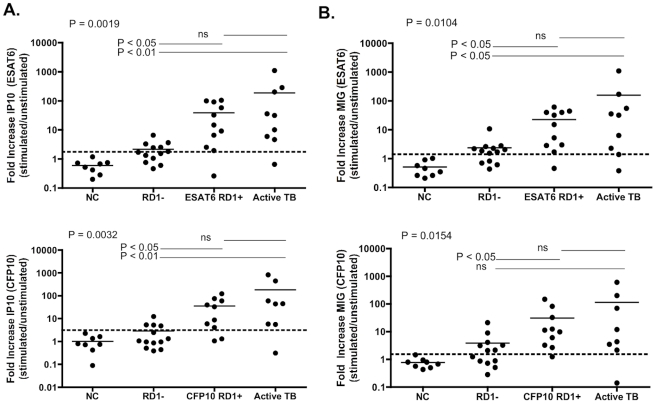
Mycobacterium tuberculosis infection as an example application. qPCR fold increase of IP10 (a) or MIG (b) in response to 8 µg/ml ESAT-6 or CFP-10 antigenic stimulation of 10^6^ fresh PBMC for 16 hours. Patients are grouped according to RD1 Elispot status or culture confirmed active TB. NC (Negative Control)  =  HIV negative individuals who are RD1 Elispot negative and negative by PPD skin test, RD1−  =  HIV positive individuals who are RD1 Elispot negative with no symptoms of active TB, RD1+  =  HIV positive individuals who are RD1 Elispot positive with no symptoms of active TB. One-way ANOVA with Kruskal-Wallis test was p = 0.0019 for IP10/ESAT-6, p = 0.0032 for IP10/CFP-10, p = 0.0104 for MIG/ESAT-6, and p = 0.0154 for MIG/CFP-10. Dunn's Multiple comparison test was then performed. p<0.05 was considered significant. The Dunn's Multiple comparisons test showed a significant difference between RD1 negative and RD1+, and RD1 negative and active TB.

As a result of these findings we decided to assess if there was a difference between MIG and IP10 expression in freshly isolated PBMCs from individuals with and without active TB. Performing the assay on fresh cells does not substantially improve the differentiation of the 3 groups of individuals observed from frozen samples. Figure 3a shows IP10 increase following both ESAT-6 and CFP-10 stimulation, while figure 3b shows MIG increase following both ESAT-6 and CFP-10 stimulation. One-way ANOVA with Kruskal-Wallis test was p = 0.0019 for IP10/ESAT-6, p = 0.0032 for IP10/CFP-10, p = 0.0104 for MIG/ESAT-6, and p = 0.0154 for MIG/CFP-10. Dunn's Multiple comparison test was then performed. The data indicate that our novel assay is able to differentiate between people that are RD1-Elispot negative (i.e. those we presume to be uninfected) and those we have categorized as latently infected (RD1 positive) (p<0.05 (IP10/ESAT-6), p<0.05 (IP10/CFP-10) (figure 3a) and (p<0.05 (MIG/ESAT-6), p<0.05 (MIG/CFP-10) (figure 3b)). However, like the IGRAs, there was no significant difference in response between RD1 positive (presumed latently MTB infected) and those with active TB disease. The qPCR approach was (with the exception of MIG/CFP-10) able to differentiate between RD1-Elispot negative and those with active TB (p<0.01 (IP10/ESAT-6), p<0.01 (IP10/CFP-10) (figure 3a) and (p<0.05 (MIG/ESAT-6) (figure 3b)).

Interestingly, even using a stringent cut-off of mean + 3SD, 10 out of 26 (38.5%) RD1 Elispot negative individuals were qPCR positive, 20 out of 20 RD1 (100%) Elispot positive individuals were qPCR positive, and 14 of our 23 (60.8%) individuals with culture-confirmed active TB are qPCR positive (table 1). Interestingly, of the 9 individuals with culture-confirmed TB that had both the Elispot and qPCR assay performed on fresh PBMC, 8 out of 9 (89%) of individuals were positive by qPCR while 7 out of 9 (78%) of these individuals were positive by Elispot (table 2). These data indicate that the novel assay is at least as sensitive in detection of TB-specific responses as the Elispot and may reveal a subset of individuals with ex vivo responses below the level of conventional detection. It is clear that T cell populations can exist below the level of detection by ex vivo Elispot using a cultured Elispot approach, so it likely that we are picking up similar low frequency populations [20]. A statistically significant difference between the qPCR fold increase observed in responses in individuals who were RD1 Elispot positive compared to those who were RD1 Elispot negative (p = 0.0016) (figure S1).

**Table 1:** qPCR was performed on frozen PBMCs from 69 individuals (grouped based on their RD1 IFN-γ Elispot status in the absence of symptoms of active TB) or clinical diagnosis of active TB.

Cut off	Patient Group	# Positive qPCR Responses					Total qPCR Positive	Patient Total
		0	1	2	3	4		
Mean +3SD	RD1−	16	7	3	0	0	10	26
	RD1+	0	7	6	3	4	20	20
	Active TB	9	3	3	3	5	14	23
	Total	25	17	12	6	9	44	69

The number of positive qPCR responses are shown per patient group. To be considered positive for MTB infection by qPCR, MIG and IP10 expression in response to IFN-γ stimulation had to be greater than all of the cutoffs, AND be positive for ESAT-6 and/or CFP-10 by MIG and/or IP10.

**Table 2:** qPCR detects a larger proportion of individuals with active TB than does RD1 Elispot in both fresh and frozen PBMC samples.

		# of Positive Patients / # of Patients Tested	
Patient Group		qPCR	RD1 Elispot
Active TB	Fresh PBMC	8/9 (88.8%)	7/9 (78%)
	Frozen PBMC	14/23 (60.8%)	6/15 (40%)

**Figure 4:**
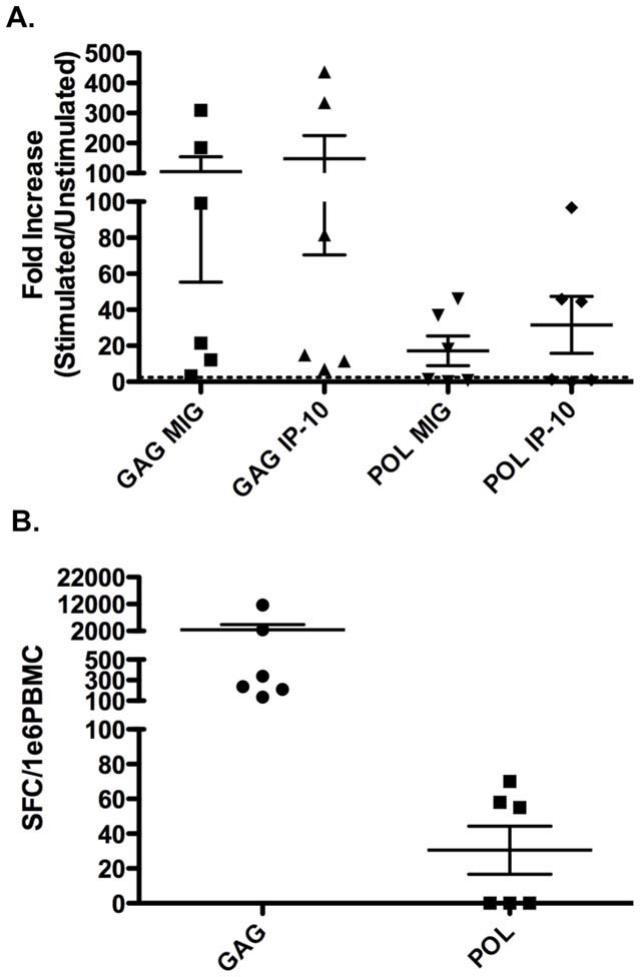
HIV infection as an example application. Fresh PBMC from 6 HIV infected individuals were stimulated with HIV Gag (left) and Pol (right) peptide pools. Strong qPCR (a) and Elispot (b) responses were observed following peptide stimulation.

Since the cohort was co-infected, and T cell responses to HIV are an important biomarker in vaccine development, we also evaluated the assay for detection of HIV-specific T cell responses. Unlike TB, these responses are largely derived from CD8+ T cells. To evaluate the use of the novel assay approach in HIV we tested fresh PBMC from 6 HIV infected individuals stimulated with HIV Gag and Pol peptide pools. Figure 4a shows that strong IP10 and MIG responses were observed following stimulation with Gag and, to a lesser extent, Pol. All individuals had a positive response following stimulation with Gag peptide pools, while only 3 out of 6 had a positive response following stimulation with Pol peptide pools. Figure 4b displays the Elispot data for the 6 HIV positive individuals. All 6 individuals had a positive Elispot response following stimulation with Gag peptide pools, while only 3 out of 6 were positive following stimulation with Pol peptide pools and, in parallel to the MIG and IP10 qPCR results, these responses were at lower spot forming cells (SFC) frequencies than for Gag responses (Gag vs Pol; MIG, IP10 and Elispot all p = 0.03).

### Quantitative Correlation between Elispot and qPCR assay

**Figure 5:**
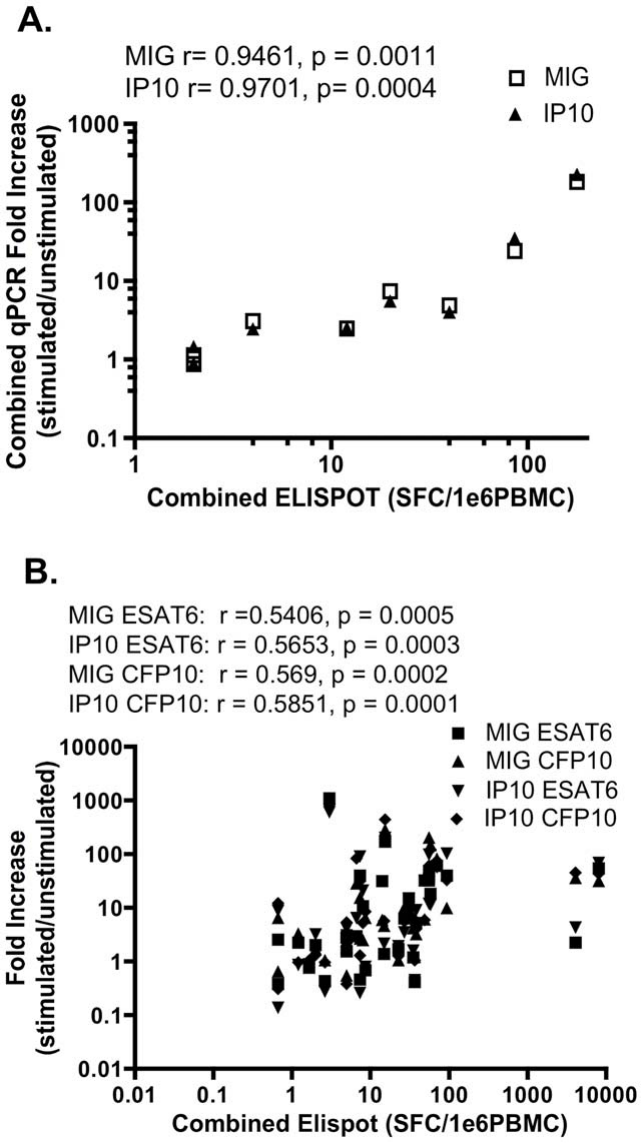
Quantitative Correlation between Elispot and qPCR assay. Correlation between MIG/IP10 qPCR and IFN-γ Elispot for CMV infection (a). The Elispot and qPCR responses to individual peptide pools were pooled and compared. Elispot SFC and qPCR fold increase of MIG and IP10 expression were significantly correlated. Correlation between MIG/IP10 qPCR and IFN-γ Elispot for *Mycobacterium tuberculosis* infection (b). *r* and *p* values shown; p<0.05 is significant. Data shown is taken only from individuals where both assays were performed on freshly isolated PBMC samples.

For such an assay to be valuable it needs not only to be sensitive but also to provide an estimate of the size of the immune response. To do this we related Elispot response to qPCR response for the pathogens studied. Figure 5a demonstrates the correlation between the CMV MIG/IP10 qPCR and CMV Elispot assay. Elispot SFC and qPCR fold increase of MIG and IP10 expression were strongly correlated following stimulation with CMV lysate (MIG: r = 0.9461, P = 0.0011; IP10: r = 0.9701, P = 0.0004). Figure 5b demonstrates the correlation between the MTB qPCR assay and the MTB RD1-specific Elispot assay. Elispot SFC and qPCR fold increase of MIG and IP10 expression were strongly correlated following stimulation with both ESAT-6 and CFP-10 (MIG ESAT-6: r = 0.5406, P = 0.0005; IP10 ESAT-6: r = 0.5653, P = 0.0003, MIG CFP-10: r = 0.569, P = 0.0002; IP10 CFP-10: r = 0.5851, P = 0.0001).

### Impact of HIV-induced immunosuppression on the MIG/IP10 qPCR assay

Next, we determined if the qPCR assay is impaired by varying levels of immunosuppression related to HIV progression. We did not find a significant correlation between the qPCR fold increase and CD4 count for MIG or IP10 on assays using frozen PBMC with active or latent disease, or using fresh cells (figure S2). These results indicate that the qPCR assay is not affected by HIV-mediated immunosuppression.

### Validation of the qPCR assay using whole blood

**Figure 6:**
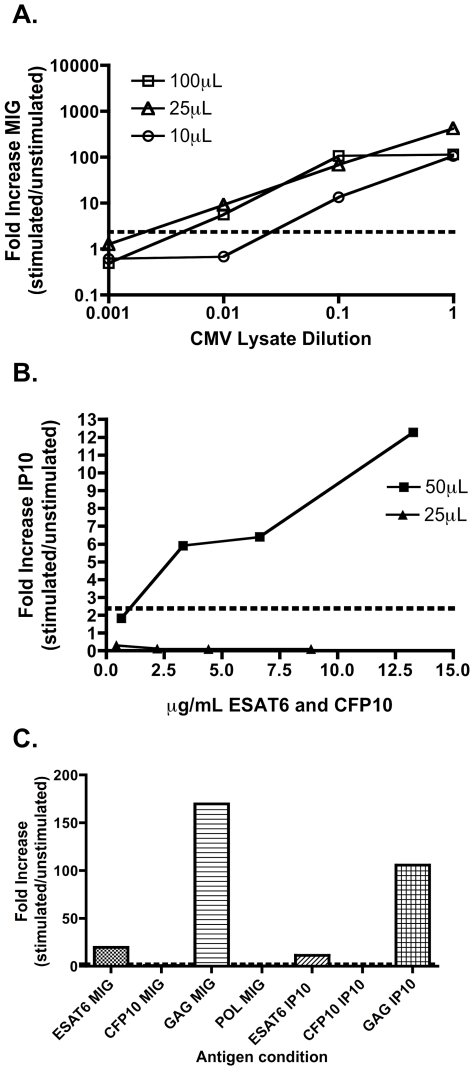
Validation of the qPCR assay using whole blood. qPCR for MIG and IP10 can detect CMV (a) MTB (b) and HIV (C) -specific immune responses using only 25 (for CMV) and 50 uL (for HIV/MTB) of whole blood. 100, 25 and 10 uL of whole blood from healthy donors with was diluted 1∶5 with R10 media and stimulated with 10 ng/mL IFN-γ or ten-fold dilutions of CMV whole cell lysate (10 uL/ml)(Sigma) for 16 hours (a). 50 and 25 ul of whole blood diluted 1∶5 with R10 media were stimulated with 1, 5, 10, or 20 µl of 0.16 mg/ml ESAT-6 and CFP-10 peptide pools for 16 hours (b). Representative example of 50 µl of whole blood taken from a HIV positive patient without symptoms suggestive of active TB and an RD1 positive Elispot, diluted 1∶5 with R10 media and stimulated with ESAT-6 and CFP-10 (both at final concentrations of 8 ug/ml) and HIV peptide pools (at a final concentration of 10 ug/ml).

Analysis of antigen-specific responses in whole blood provides a significant methodologic advantage and in particular retains an additional population of reporter cells in the form of neutrophils (which are lost during PBMC separation). We therefore confirmed that the assay could be successfully performed on whole blood to detect antigen-specific T cells. We analyzed the sensitivity of the whole blood assay using limiting quantities of blood to identify T cell responses *ex vivo*. Figure 6a shows that the qPCR assay performs well when whole blood is stimulated with CMV antigens. We found that we could reliably reduce blood volumes to as low as 25 µL per sample and still retain high sensitivity and specificity, in particular for IP10 detection. Figure 6b shows that 50 µL of blood is more suitable when whole blood (from an HIV positive individual with presumed latent MTB infection) is stimulated with the RD1 antigens ESAT-6 and CFP-10 (data shown is representative of 8 examples). Figure 6c displays data from stimulation of 50 µL of whole blood from an HIV infected individual with presumed latent MTB infection with a selection of peptide pools including HIV Gag and Pol. Strong responses are observed following gag and ESAT-6 stimulation.

## Discussion

In this study, we developed a novel assay for the detection of antigen-specific T cells. We then assessed its use for the detection of MTB infection in a HIV-coinfected cohort. This report demonstrates that qPCR using IFN-γ reporter genes can be used to accurately and sensitively detect antigen-specific CD4+ and CD8+ T cell responses against persistent infection, e.g. CMV, MTB and HIV. Importantly, we show that such an assay can be performed on small quantities of whole blood without loss of accuracy, and can be performed on immunocompromised individuals.

Other studies have investigated MIG and IP10 as a marker of CMV, MTB and other infections. Such analyses have used Northern Blotting, ELISA, Elispot, FACs, and multiplex to measure IP10 and MIG concentration in patient serum or production in response to antigens. These studies indicated that MIG and IP10 can be used as a surrogate for IFN-γ production and that their upregulation is in an antigen-specific manner. However, measuring MIG and IP10 protein production with these techniques did not compare to the sensitivity of the standard IFN-γ Elispot. This novel qPCR assay approach, reported here, appears to match and even surpass the sensitivity assay of the Elispot platform. T cell populations can exist below the level of detection of the ex vivo Elispot using a cultured Elispot approach, so it likely that the qPCR assay described here are picking up similar low frequency populations[20]. It should be noted that there was no obvious diagnostic benefit of stimulating separately with both ESAT-6 and CFP-10 antigens and therefore we propose that future assays stimulate with combined peptide pools.

The qPCR platform could be of significant value for a number of reasons. Firstly, the ability to evaluate T cell responses with high sensitivity using only 25–50 uL of blood means patient sampling could take place in many more clinical settings, where such volumes would be easily obtainable using a fingerprick. This includes field studies, population screening and pediatric populations, although clearly large scale clinical studies would be required to compare the utility of such a test to established methodologies. Secondly, the whole blood qPCR methodology could be easily implemented in a number of labs, where the cellular immunological techniques required for e.g. Elispot are not available. Thirdly, the assay is highly flexible and can be stored at any stage after stimulation for batching. Unlike direct assays like the Elispot, the cDNA may also be reprobed for checking of data, or potentially for testing of novel antigen-dependent transcripts. Finally, the ability to test both MIG and IP10 with high sensitivity means that one chemokine could be used as the main screening test, with the second as a confirmatory test if necessary. Although we have focused on T_H_1 responses, the same approach could also be applied using different reporters to the sensitive detection of antigen-specific T_H_2 cells involved in allergy (e.g. IL-4 or 5 induced genes) and IL-10 or IL-17 secreting T cells. Even for Th1 responses it is likely that there are other transcriptional signals which could be identified which may surpass the sensitivity and specificity of MIG and IP10 in whole blood and/or PBMC and a genome-wide approach is in progress; by comparison, analysis of IFNg upregulation by qPCR showed only a limited response (typically <2 fold, data not shown), likely due to dilution of the signals.

In conclusion, we provide evidence here for a novel approach to the measurement of antigen specific T cells in human populations. The qPCR assay approach is sensitive, robust and can be performed on fingerprick quantities of whole blood. The technique's broad applicability and improved sensitivity would be highly useful in studying infections such as Hepatitis C Virus where T cell responses are at or below threshold detection of conventional assays and evaluating the efficacy of T cell inducing vaccines, such as those currently in trials for malaria, TB and HIV. This assay therefore possesses the potential to meet the urgent need for simple and robust assays for T cell function.

## Supporting Information

Figure S1 **Higher MIG and IP10 expression in RD1 Elispot positive individuals.** MIG and IP10 expression as measured by qPCR is significantly higher in patients who are also positive by RD1 Elispot (P = 0.0016, Mann-Whitney t-test) than in patients who are negative by RD1 Elispot.
(TIFF)

Figure S2 **qPCR assay is not affected by HIV-mediated immunosuppression.** MIG and IP10 production as measured by qPCR is not affected by CD4 T cell count in frozen or fresh PBMCs. MIG and IP10 expression in response to RD1 antigens ESAT-6 and CFP-10 from thawed PBMCs from Active TB patients (a), thawed PBMCs from non-active (RD1+ and RD1-, active TB excluded) (b) and fresh PBMCs of non-active and active TB patients (c). Correlation was insignificant (p>0.05) for all of the analyses.
(TIFF)
