# Supplementary figures and images for: A Molecular Assay for Sensitive Detection of Pathogen-Specific T-Cells
Source: PLoS One. 2011 Aug 11;6(8):e20606. doi: 10.1371/journal.pone.0020606 (PMC3154901; doi:10.1371/journal.pone.0020606)

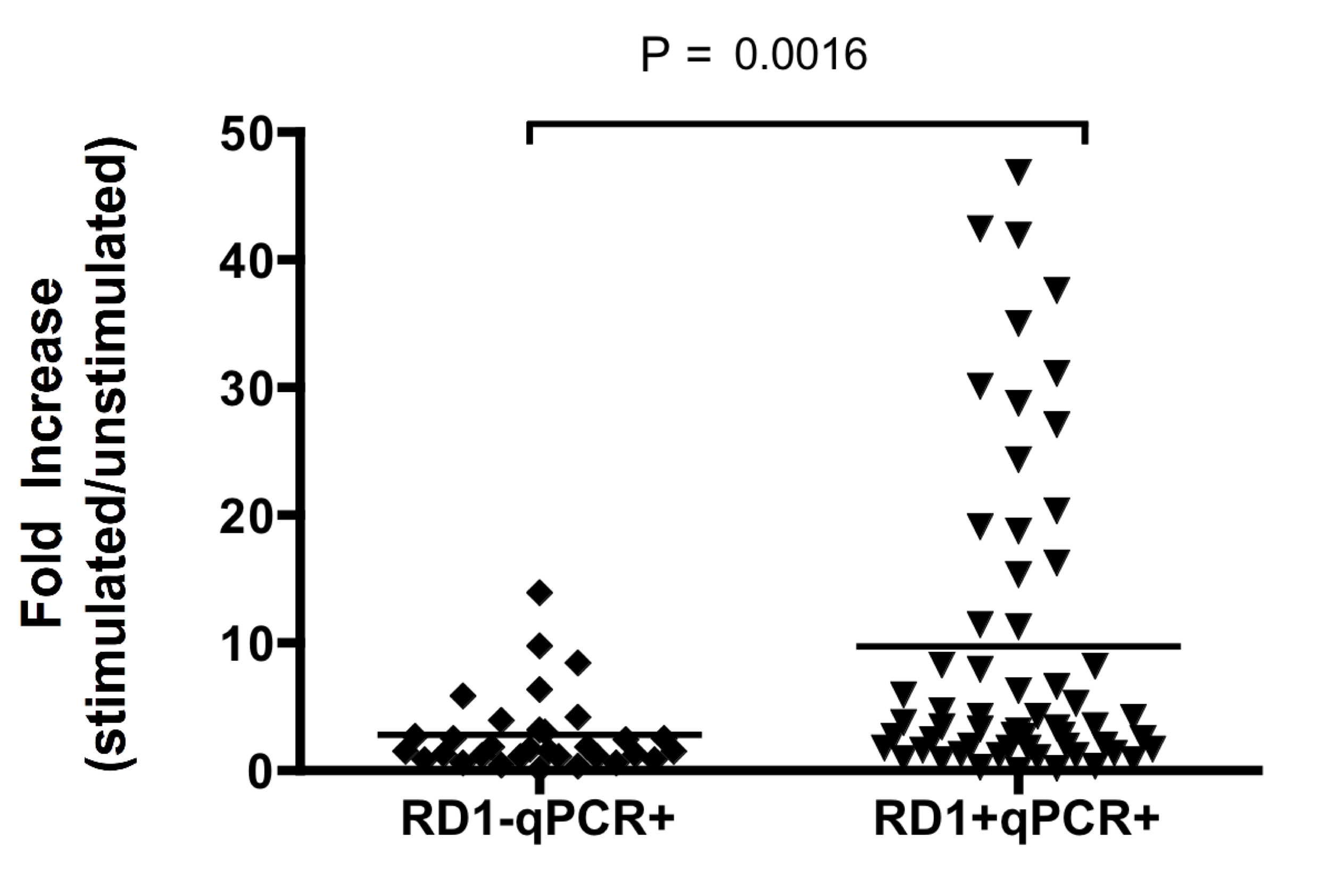

Supplement: Figure S1 — Higher MIG and IP10 expression in RD1 Elispot positive individuals. MIG and IP10 expression as measured by qPCR is significantly higher in patients who are also positive by RD1 Elispot (P = 0.0016, Mann-Whitney t-test) than in patients who are negative by RD1 Elispot. (TIFF) [file pone.0020606.s001.tiff]

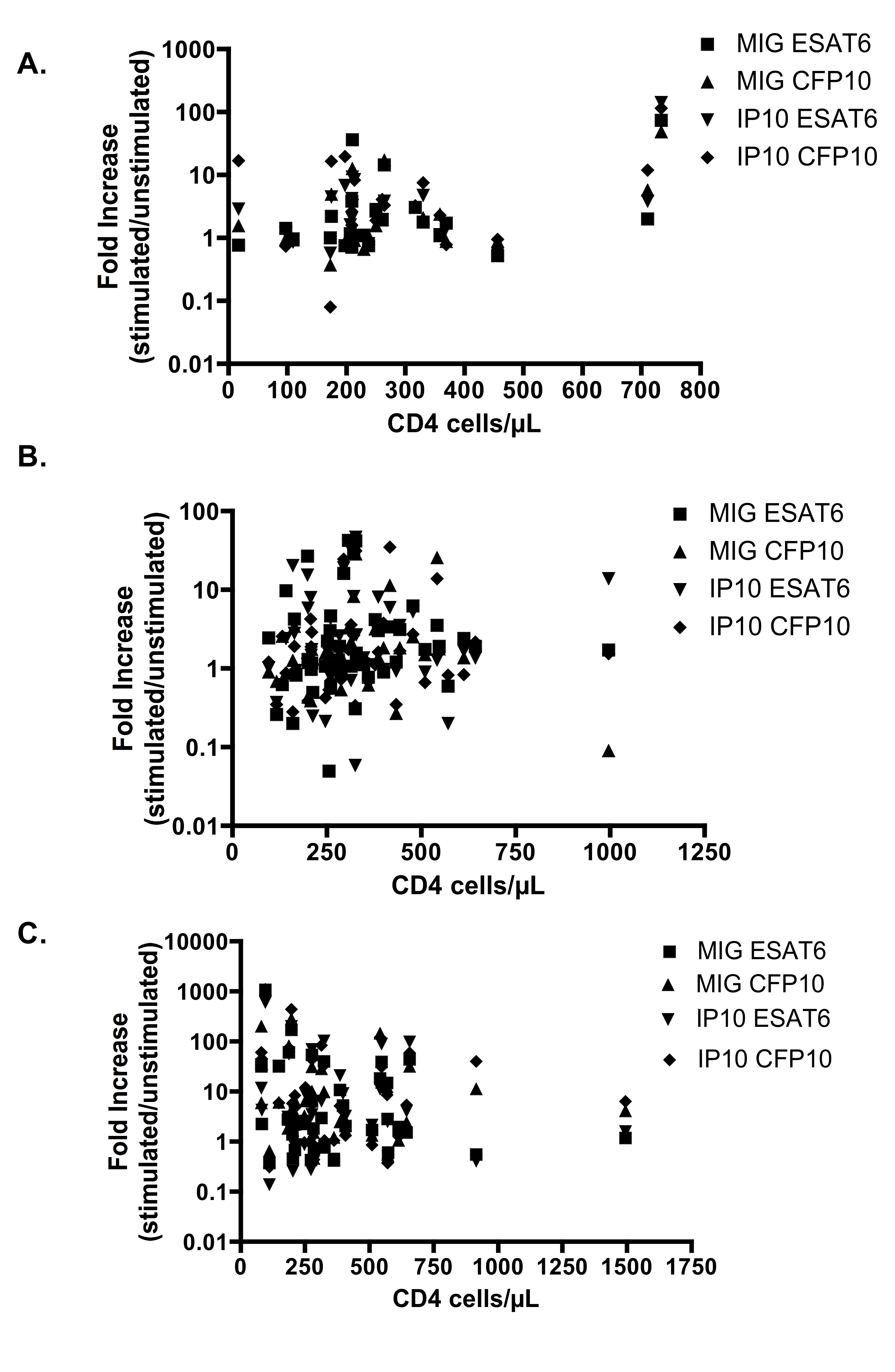

Supplement: Figure S2 — qPCR assay is not affected by HIV-mediated immunosuppression. MIG and IP10 production as measured by qPCR is not affected by CD4 T cell count in frozen or fresh PBMCs. MIG and IP10 expression in response to RD1 antigens ESAT-6 and CFP-10 from thawed PBMCs from Active TB patients (a), thawed PBMCs from non-active (RD1+ and RD1-, active TB excluded) (b) and fresh PBMCs of non-active and active TB patients (c). Correlation was insignificant (p>0.05) for all of the analyses. (TIFF) [file pone.0020606.s002.tiff]
